# Development and Evaluation of a Multiplexed Immunoassay for Simultaneous Detection of Serum IgG Antibodies to Six Human Coronaviruses

**DOI:** 10.1038/s41598-018-37747-5

**Published:** 2019-02-04

**Authors:** Suvang U. Trivedi, Congrong Miao, Joseph E. Sanchez, Hayat Caidi, Azaibi Tamin, Lia Haynes, Natalie J. Thornburg

**Affiliations:** 1 Oak Ridge Institute for Science and Education, Oak Ridge Associated Universities, 100 ORAU way, Oak Ridge, TN 37830 USA; 2 National Center for Immunization and Respiratory Diseases, Division of Viral Diseases, Gastroenteritis and Respiratory Viruses Laboratory Branch, Centers for Disease Control and Prevention 1600 Clifton Rd NE, Atlanta, GA 30329 USA

## Abstract

Known human coronaviruses (hCoV) usually cause mild to moderate upper-respiratory tract illnesses, except SARS-CoV and MERS-CoV, which, in addition to mild illness can also be associated with severe respiratory diseases and high mortality rates. Well-characterized multiplexed serologic assays are needed to aid in rapid detection and surveillance of hCoVs. The present study describes development and evaluation of a multiplexed magnetic microsphere immunoassay (MMIA) to simultaneously detect immunoglobulin G (IgG) antibodies specific for recombinant nucleocapsid proteins (*rec*N) from hCoVs 229E, NL63, OC43, HKU1, SARS-CoV, and MERS-CoV. We used paired human sera to screen for IgG with reactivity against six hCoVs to determine assay sensitivity, specificity and reproducibility. We found no signal interference between monoplex and multiplex assay formats (R^2^ range = 0.87–0.97). Screening of paired human sera using MMIA, resulted in 92 of 106 (sensitivity: 86%) as positive and 68 of 80 (specificity: 84%) as negative. This study serves as a proof of concept that it is feasible to develop and use a multiplexed microsphere immunoassay as a next generation screening tool for use in large scale seroprevalence studies of hCoVs.

## Introduction

Coronaviruses (CoVs) belong to *Coronaviridae* family in the order of *Nidovirale*^1^. They are single-stranded, positive-sense RNA viruses with an outer envelope and crown-like morphologies, formed by spike (S) attachment glycoproteins^2^. Based on phylogenetic analysis, all CoVs are classified into four genera, *Alpha-*, *Beta-*, *Gamma*-, or *Deltacoronavirus*^3^. Currently, there are six strains of human coronaviruses (hCoVs), known to cause mild to severe, upper and lower respiratory tract infections in humans^2^. From early 1960s to early 2000s, there were only two hCoVs known to cause infections in humans, 229E (*Alphacoronavirus*) and OC43 (*Betacoronavirus*)^4^. In 2003, an outbreak of severe acute respiratory syndrome (SARS)-CoV (*Betacoronavirus*), originating from Guangdong province in China, resulted in 8,096 reported cases and 774 deaths worldwide^5^. Following this outbreak, hCoV-NL63 (*Alphacoronavirus*) and hCoV-HKU1 (*Betacoronavirus*)^6^ were identified as sources of upper respiratory and gastrointestinal tract infections in hospitalized patients^7^. More recently, MERS-CoV (Middle East respiratory syndrome coronavirus) has been identified as the etiological agent for an ongoing epidemic of severe respiratory infection with high mortality rate in the Middle East^5^.

There are no vaccines or antivirals approved for the treatment or prevention of infections caused by SARS-CoV or MERS-CoV^8^. In the absence of virus-specific control measures, the key to controlling a reemergence of SARS-CoV and spread of MERS-CoV, is rapid detection and isolation of suspect cases. Large scale, population-based seroprevalence studies are very useful for identification of critical control points in disease outbreaks as they provide important data about susceptible populations and potential of future outbreaks through predictive modeling^9^. Use of purified or recombinant viral antigen based enzyme-linked immunosorbent assays (ELISAs) coupled with molecular detection assays is an effective approach in population based surveillance studies^10^. We have developed and validated hCoV recombinant nucleocapsid protein (*rec*N) indirect ELISAs that individually detect immunoglobulin G (IgG) antibodies to all six hCoVs.

The hCoV nucleocapsid (N) protein is abundantly expressed during infection and is, therefore, one of the ideal candidate antigens for the development of microtiter plate based ELISAs^11^. However, traditional plate ELISAs are limited to detecting IgG antibodies to a single hCoV *rec*N antigen per well. In contrast, a magnetic bead-based multiplexed immunoassay (MMIA) would allow for the simultaneous detection of IgG antibodies to all six hCoV *rec*N in a single test well, reducing sample consumption. In addition, multiplexing would allow higher sample throughput at lower reagent, labor, and material costs, without losing sensitivity and specificity of traditional ELISAs. The present work describes the development and validation of a MMIA to detect human serum IgG against six hCoV *rec*N as a next generation screening tool in population-based seroprevalence studies.

## Results

### Titration of Antigen Concentration

**Figure 1:**
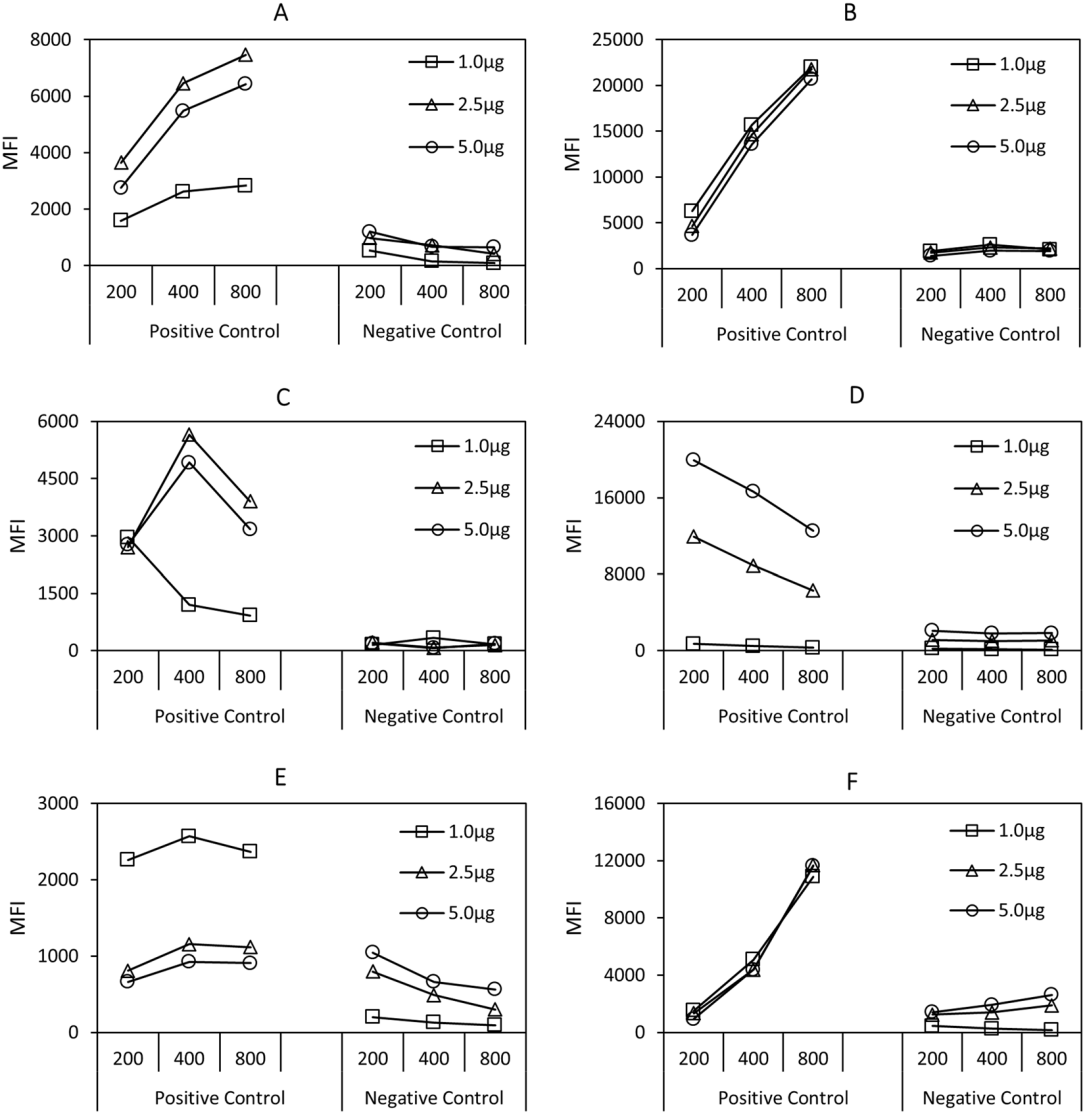
Titration of recombinant nucleocapsid (*rec*N) proteins of hCoVs (**A**) 229E, (**B**) NL63, (**C**) OC43, (**D**) HKU1, (**E**) SARS-CoV and (**F**) MERS-CoV at concentrations of 1.0 µg (□), 2.5 µg (∆), and 5.0 µg (○) per conjugation reaction. Positive and negative controls for each hCoV were tested at dilutions 1:200, 1:400 and 1:800. Mean Fluorescence Intensity (MFI) values of negative control antigens (pET *E. coli* and His-BoNT) were subtracted from the MFI values of corresponding positive control antigen for each antigen.

A monoplex assay for each hCoV *rec*N conjugated bead set was developed and standardized to determine optimum antigen quantity per bead coupling reaction, optimum serum dilution and assay cut-off mean fluorescent intensity (MFI) values. Each bead set was conjugated with 1.0 µg, 2.5 µg, or 5.0 µg of antigen per 100 µl of conjugation reaction (1.25 × 10^6^ beads). A checkerboard titration assay was carried out for each antigen against a series of 2-fold (1:100–1:800) positive and negative control sera. The optimum working antigen amounts were determined to be 1.0 µg for SARS-CoV *rec*N, His-BoNT, NL63 *rec*N; 2.5 µg for 229E *rec*N, MERS-CoV *rec*N, OC43 *rec*N3; pET *E. coli*, and 5.0 µg for HKU1 *rec*N per conjugation reaction (1.25 × 10^6^ beads) (Fig. 1). Based on positive serum control titration results, optimum dilutions for each antigen were determined to be 1:400 for OC43, NL63, HKU1 and SARS-CoV; and 1:800 for 229E, and MERS-CoV (Fig. 1). The optimum antigen quantities and positive control serum dilutions were selected based highest signal to background ratio, most reproducible results and economical usage of antigen stocks.

### Correlation between monoplex and multiplex assay

**Figure 2:**
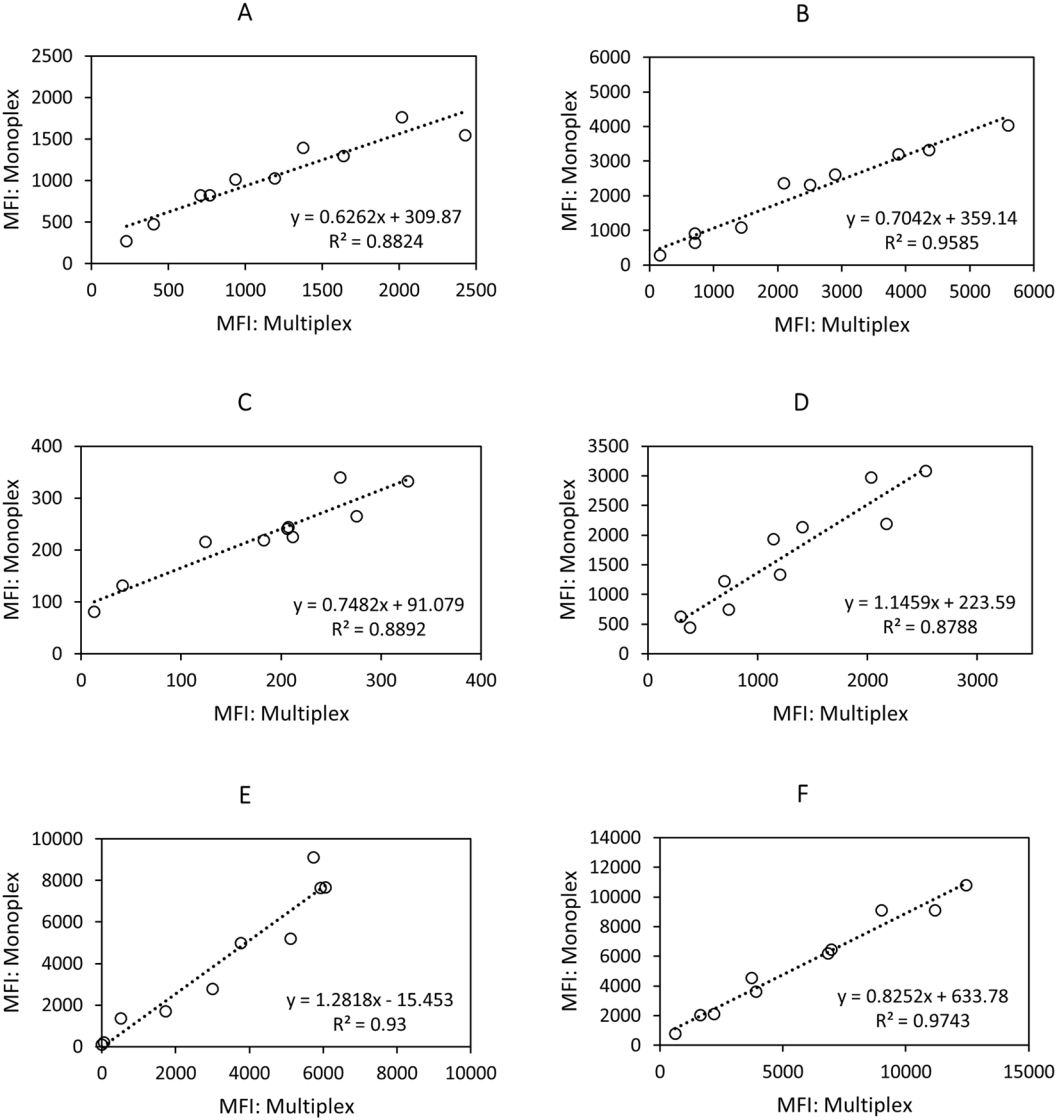
Correlation of Mean Fluorescence Intensity (MFI) values between monoplex and multiplex microsphere immunoassays using positive control serum for (**A**) 229E, (**B**) NL63, (**C**) OC43, (**D**) HKU1, (**E**) SARS-CoV and (**F**) MERS-CoV.

We compared the MFI values generated by monoplex assays with multiplex assay for each antigen to investigate possible loss of sensitivity due to drop in MFI values. Positive control serum pools for each hCoV were serially diluted in 2-fold dilutions (1:100–1:51200) and screened using monoplex and multiplex microsphere immunoassays. In each assay format, 200 beads (100 beads in duplicate) were counted for each *rec*N conjugated bead set. Linear regression analysis was performed to determine correlation coefficient for each *rec*N antigen. For all *rec*N bead sets, statistically significant correlations were observed between the monoplex and multiplex formats with a correlation coefficient (R^2^) ranging from 0.88 to 0.97 (Fig. 2).

### Sensitivity, Specificity and cut-off MFI values

To determine the sensitivity, specificity and cut-off MFI values for each antigen, receiver operating characteristic (ROC) analysis was carried out based on screening of human sera collected from individuals whose respiratory specimens were tested by reverse real time polymerase chain reaction (rRT-PCR) for presence or absence of hCoV infections. Acute and convalescent phase sera from individuals tested positive for hCoVs 229E, NL63, OC43, and HKU1 infections as well as convalescent phase sera, from confirmed positive infections of MERS-CoV and SARS-CoV were screened using MMIA. Additionally, paired sera, from individuals tested negative for hCoV infection were also screened using MMIA. Percentage sensitivity (%Se) and specificity (%Sp) for each *rec*N was estimated over a wide range of cut-off MFI values and plotted as Two Graph – Receiver Operating Characteristic curve (TG-ROC) using SPSS software (Data not shown). The MFI value at which the paired values of (%Se) and (%Sp) were optimal (intersection of %Se and %Sp graph lines) were selected as the cut-off MFI for respective *rec*N antigen (data not shown). The cut-off MFI values of 1333, 513, 228, 1171, 1333 and 520 were selected for 229E, NL63, OC43, HKU1, SARS-CoV and MERS-CoV *rec*N monoplex assays, respectively. The corresponding sensitivity and specificity values are described in Table 1.

**Table 1:** Diagnostic sensitivity and specificity of all six hCoV *rec*N in *Luminex* multiplex microsphere immunoassay in relation to cut-off MFIs, and %sensitivity and %specificity determined after ROC analysis of known positive and negative sera.

	Cut-off MFI	%Sensitivity (Pos/Total)	%Specificity (Neg/Total)
229E *rec*N	1333	100 (8/8)	88.75 (71/80)
NL63 *rec*N	513	83.33 (15/18)	83.75 (67/80)
OC43 *rec*N3	228	80.95 (34/42)	81.25 (65/80)
HKU1 *rec*N	1171	89.28 (25/28)	86.25 (69/80)
MERS-CoV *rec*N	1333	85.71 (6/7)	95.00 (76/80)
SARS-CoV *rec*N	520	80.00 (4/5)	85.00 (68/80)

**Figure 3:**
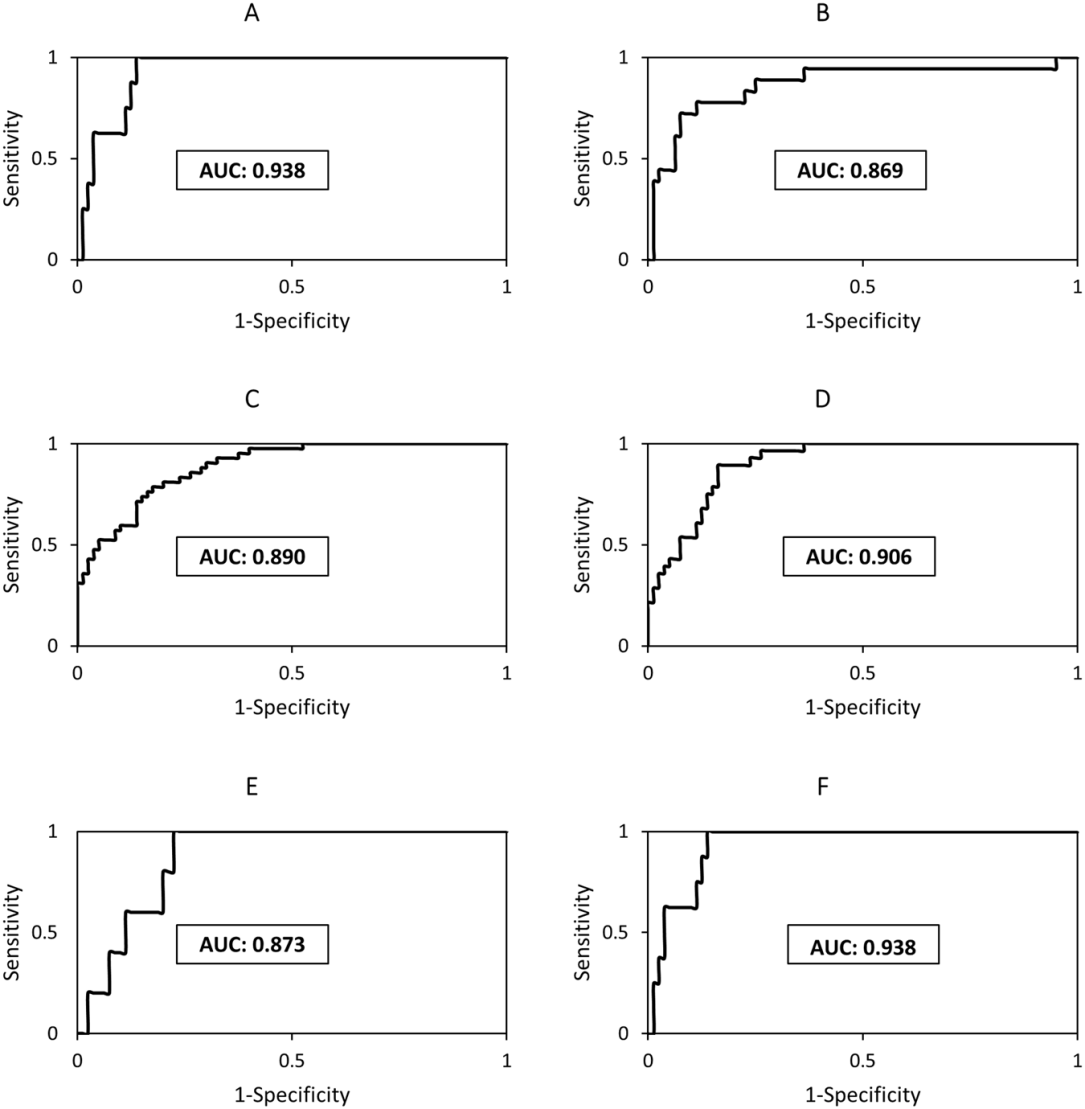
Receiver Operating Characteristic curve (ROC) analysis to determine area under the curve (AUC) values for (**A**) 229E, (**B**) NL63, (**C**) OC43, (**D**) HKU1, (**E**) SARS-CoV and (**F**) MERS-CoV *rec*N in MMIA using real-time RT-PCR as the reference diagnostic test.

To determine the overall assay performance of MMIA, areas under curves (AUCs) were calculated for all six hCoV *recN* using RT-PCR as the reference diagnostic test (Fig. 3). The calculated AUC values for 229E, NL63, OC43, HKU1, SARS-CoV, and MERS-CoV *rec*N, were 0.938, 0.869, 0.890, 0.906, 0.873 and 0.913, respectively (Fig. 3), indicating moderate to high level of test accuracy.

### Reproducibility of Multiplex Microsphere Immunoassay

To assess the reproducibility of the MMIA, a panel of positive control serum samples for each hCoV was run in duplicate at optimum serum dilutions on two different plates on the same day and was repeated with a new batch of conjugated beads on a different day. Coefficients of variation (CV) for each replicate for each antigen was calculated and averaged (Table 2). All assays except NL63 *rec*N gave CV values below 10% indicating MMIA precision.

**Table 2:** Assay reproducibility of the *Luminex* multiplex microsphere immunoassay.

	Within plate %CV	Between plates %CV	Between assays %CV
	(n = 24)	(n = 48)	(n = 94)
229E	2	2	6
NL63	5	12	23
HKU1	2	3	6
OC43	6	4	6
SARS-CoV	8	8	6
MERS-CoV	2	2	3

%CV = percent coefficient of variation.

### Cross-reactivity between recN antigens

**Figure 4:**
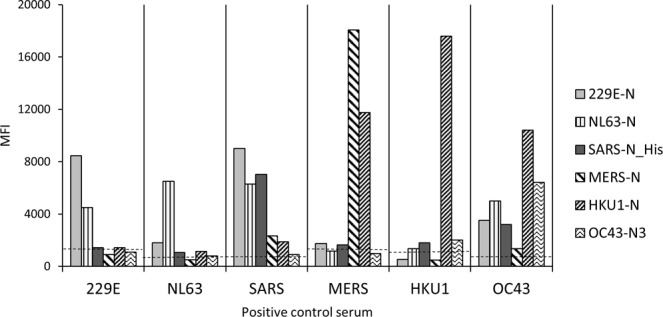
Cross reactivity of hCoV *rec*N conjugated beads with Positive control serum samples for all six hCoVs in a multiplex microsphere immunoassay (MMIA) @ 1:400 dilution.

To examine cross reactivities between all hCoV *rec*N in human sera, we screened each positive control of hCoV in a multiplex format at dilutions ranging 1:100–1:800. The results show overall minimum cross reactivity amongst hCoV *rec*N (Fig. 4). However, positive controls of the hCoVs of the same antigenic groups showed cross reactivity with each other. Positive controls of group 1 (alpha) human coronaviruses, 229E and NL63, showed reactivity with each other but not with group 2 hCoVs. However, SARS-CoV (group 2b) positive control serum reacted with group 1 CoVs, 229E and NL63. Positive control serum for OC43, a group 2a (beta) coronavirus, reacted with HKU1 *recN*, another member of group 2a whereas positive control for MERS-CoV (group 2c) reacted with HKU1 *rec*N.

## Discussion

Use of bead-based multiplexed immunoassay technology has been gaining recognition as a next generation screening tool in seroprevalence studies. Several research groups have reported the development of bead-based multiplexed immunoassays to detect antibodies against a variety of human viral pathogens. A duplex assay was developed to detect IgG against recombinant nucleocapsid proteins of human respiratory syncytial virus (hRSV) and human metapneumovirus (hMPV)^10^. A quadruplex assay was developed to detect IgGs against measles, mumps, rubella and varicella-zoster virus^12^. A thirteen-plexed immunoassay has been developed to detect IgM and IgGs for serodetection of arboviruses^13^. The results of the current study shows that it is feasible to use a multiplexed bead immunoassay to screen human sera for IgGs against the six human coronavirus recombinant nucleocapsid proteins.

The use of ROC analysis in our study not only facilitated selection of optimal MFI cut-off values, but also helped determine the accuracy and usefulness of our test method by calculating the area under the ROC curves^14^. A perfect test has an AUC of 1 and a useless test has an area of 0.5, the equivalent of a coin toss. Based on arbitrary criteria, the following guidelines have been suggested for interpretation of AUC values in context of test accuracy: low (0.5 < AUC ≤ 0.7), moderate (0.7 < AUC ≤ 0.9), and high (0.9 < AUC ≤ 1)^15^. Based on our assay test results, the calculated AUC ranged from 0.87 to 0.94, suggesting a high to high-moderate levels of accuracy and overall assay performance. The cross reactivity experiment results showed positive control of SARS-CoV reacting with group 1 hCoVs 229E and NL63 *recN*. A major limitation of these assays is that we are unable to distinguish the difference between cross reactivity and evidence of previous infections. SARS-CoV *recN* has been shown to react with polyclonal antisera of group 1 coronaviruses in a western blot analysis^16^. However, this cross reactivity was one-way from SARS-CoV to other hCoV *recN* in this study which has also been previously confirmed by Che *et al*., in a separate study^17^. The multiple sequence identity matrix of all hCoV nucleocapsid protein sequences show amino acid homology ranging from 20% to 63% (data not shown). In contrast, group 1 hCoVs showed only 20% to 22% sequence homology with group 2 hCoVs which explains the lack of detectable cross-reactivity between these two antigenic groups. However, sequence homology was high between nucleocapsid sequences of OC43 and HKU1 (%) and that of HKU1 and MERS-CoV (%). Higher percentage of nucleocapsid protein sequence homology may indicate the shared epitopes in the conserved and/or overlapping regions between hCoVs of the same antigenic groups. Since the previous hCoV infection history of patients enrolled in the study was unavailable, dual infection with OC43 and HKU1 could also have contributed to the cross-reactivity, though that is unlikely for HKU1 and MERS-CoV.

Our hCoV MMIA offers high throughput testing at reduced cost; the assay can be completed in less time with less labor using less clinical specimen and assay reagents. Moreover, MMIA allows the flexibility of mixing and matching the bead sets depending upon the analysis requirements. Overall, the results of our study showed that development of a bead-based multiplex immunoassay for screening human sera for IgG antibodies against hCoV *recN* proteins is feasible and may serve as an effective tool for large scale seroprevalence studies such as examining prevalence of hCoVs in young children of different ages.

## Materials and Methods

### Human Serum Samples

All methods were carried out in accordance with CDC’s institutional review board (IRB) guidelines and regulations. All protocols were approved by CDC’s IRB. All patients were hospitalized with pneumonia symptoms, gave informed consent, and their respiratory specimens were tested by rRT-PCR to determine etiology. Paired acute and convalescent phase human sera collected from individuals whose respiratory specimens were tested positive for hCoV-229E (n = 4), hCoV-NL63 (N = 9), hCoV-OC43 (n = 21), and hCoV-HKU1 (n = 14), by rRT-PCR, were included in a positive control panel for the study. Human sera from patients with confirmed SARS-CoV (n = 5) and MERS-CoV infections (n = 7), tested during outbreak investigation by our laboratory, were also included to the positive control panel. Acute sera were collected upon hospital admission, and convalescent phase sera were obtained 3–7 weeks later. From this panel of positive sera, convalescent phase sera exhibiting ≥ 4 fold increase in IgG titers, were pooled together to create a positive control for each hCoV *rec*N. Similarly, acute and convalescent phase human sera, collected from individuals with acute respiratory illness whose respiratory specimens tested negative for all six hCoVs by real-time RT-PCR and exhibited no IgG reactivity when screened by in-house hCoV *rec*N enzyme immunoassays, were included to create a negative control panel.

### Expression and purification of recombinant nucleocapsid protein antigens

For this study, we chose to use recombinant coronavirus nucleocapsid (N) proteins. N antigens The full length SARS-CoV *rec*N and a negative control antigen, the nontoxic 50-kDa C-terminal fragment of the botulinum neurotoxin serotype A (BoNt/HcA), were expressed and purified as described previously^18^. Expression and purification of full length hCoV-229E *rec*N, hCoV-NL63 *rec*N, hCoV-HKU1 *rec*N, MERS-CoV *rec*N and purified protein from *E. coli* containing pET-28 vector without the N gene, as a negative antigen control, were carried out as described previously for hCoV-OC43 *rec*N3^19^. Briefly, RNAs of respective hCoV-229E, NL63, HKU1, and MERS-CoV nucleocapsid (N) genes were amplified by RT-PCR using following primer pairs: 229E, forward, 5′-GGATCCCATATGGCTACAGTCAAATGGGCTG-3′ and reverse, 5′-GGACTCGAGCTCTTAGTTTACTTCATCAATTATGTCAG-3′; NL63, forward, 5′-GGATCCCATATGGCTAGTGTAAATTGGGCCGATG-3′ and reverse, 5′-CTCGAATTCTTATTAATGCAAAACCTCGTTGAC-3′; HKU1 forward, 5′-CGGAATTCGATGTCTTATACTCCCGGT-3′ and reverse, 5′-TTTTCCTTTTGCGGCCGCTTAAGCAACAGAGTCTTCTA-3′; MERS-CoV forward, 5′-GGATCCCATATGGCATCCCCTGCTGCACCTCGTGCT-3′ and reverse, 5′-CTCGAATTCTTACTAATCAGTGTTAACATCAATCAT-3′. The amplified genes were cloned into a pET-28 vector encoding a C-terminal polyhistidine (His) 6 tag. Recombinant antigens were expressed by IPTG induction in *Escherichia coli* strain BL21 (DE3) cells, and were purified by metal affinity chromatography (QIAGEN, Valencia, CA). Purified proteins were analyzed by SDS-PAGE and western blots.

### ELISA

The positive and negative control human sera panels were first tested by in-house hCoV *rec*N ELISAs to determine titers of IgG antibodies against individual hCoV *rec*N. All human sera were initially tested in duplicate at 1:200 followed by titration at a 4-fold dilution range of 1:100 to 1:6400. In house indirect ELISAs for *rec*N have been developed and standardized for screening of serum samples for IgG reactivity against hCoV-229E *rec*N, hCoV-NL63 *rec*N, hCoV-HKU1 *rec*N and MERS-CoV *rec*N, using modified version of previously described indirect ELISAs for SARS-CoV *rec*N and hCoV-OC43 *rec*N3 (Haynes *et al*.,^18^; Blanchard *et al*.,^19^). Briefly, 96-well transparent flat bottom Immulon 2HB microtiter plates (Thermo Scientific, Rochester, NY) were coated with purified hCoV-229E *rec*N (6.75 ng/well), hCoV-NL63 *rec*N (5 ng/well), hCoV-OC43 *rec*N3 (25 ng/well), hCoV-HKU1 *rec*N (40 ng/well), MERS-CoV *rec*N (40 ng/well) or SARS-CoV *rec*N (12.5 ng/well) and negative control antigens *p*ET *E. coli* (5.0–40 ng/well) or His-BoNT (12.5 ng/well), diluted in sterile phosphate buffered saline (PBS, pH 7.4) and incubated overnight at 2–8 °C. The next day, plates were washed three times with 150 µl of PBS-T (PBS containing 0.05% Tween-20), and incubated with serum dilutions prepared in PBS-T-M (PBS containing 0.05% Tween-20 and 5% skim milk) for 1 h at 37 °C. Following incubations, the plates were washed three times in PBS-T and incubated with 1:4000 dilution of HRP-conjugated goat anti-human IgG (H + L, KPL, Gaithersburg, MD) prepared in PBS-T-M for 1 h at 37 °C. Following incubation, plates were washed three times with 150 µl of PBS-T, and incubated with ABTS^®^ peroxidase substrate (2,2-azino-di-(3-ethylbenzthiazoline-6-sulfonate)) (KPL, Gaithersburg, MD) at 37 °C for 30 min. The reaction was terminated by adding ABTS^®^ peroxidase stop solution (5% sodium dodecyl sulfate) and the optical density (OD) measured at 405/490 nm using a TECAN Infinity microplate reader (Mannedof, Switzerland). The OD values of the negative control wells (n) were subtracted from and divided by the OD values of antigen-coated wells (p) and the average OD values of the antigen-coated wells were calculated as the difference (p-n) and ratio (p/n).

### Conjugation of viral antigens to carboxylated magnetic beads

Purified His-tagged whole or truncated *rec*N of hCoV-229E, hCoV-NL63, hCoV-OC43, hCoV-HKU1, SARS-CoV, and MERS-CoV, along with pET *E. coli* and His-BoNT as negative antigen controls, were covalently conjugated to eight spectrally distinct MagPlex^®^ pro-magnetic microsphere (bead) sets (*Luminex*^*®*^ Corporation, Austin, TX), using the two step carbodiimide coupling protocol provided with Bio-Plex amine coupling kit (Bio-Rad Laboratories, Hercules, CA). All washing steps were performed using a magnetic separator (*Luminex*^®^). The beads were vortexed vigorously for 30 sec and sonicated for 15 sec in order to disperse the bead aggregates. For 1X scale conjugation reaction, 1.25 × 10^6^ of monodispersed beads from each bead set were washed in PBS (pH 7.4). The washed beads were activated by incubation in 100 μl of activation buffer (provided with the kit) containing freshly prepared 10 µl of 50 mg/ml N-hydroxysulfosuccinimide sodium salt (sulfo-NHS) (Pierce, Rockford, IL, USA) and 10 μl of 50 mg/ml 1-Ethyl-3-(3-dimethlyaminopropyl) carbodiimide hydrochloride (EDC) (Pierce) for 20 min at room temperature in the dark with continuous shaking. The activated beads were washed twice with PBS (pH 7.4). Coupling was performed by suspending the activated beads in 500 μl of PBS (pH 7.4) containing 1.0 µg, 2.5 µg, or 5.0 µg of hCoV *rec*N or its corresponding negative control antigen and incubation for 2 h at room temperature in dark with continuous shaking. The covalently linked beads were washed once with 500 μl PBS (pH 7.4), resuspended in 250 µl StabiliGuard02 (SG02)^®^ (Surmodics Inc., Minneapolis, MN), and incubated at 37 °C for 2 h in the dark with continuous shaking. Finally, the beads were magnetically separated and resuspended in 150 µl of SG02, enumerated using a hemocytometer and stored at 2–8 °C in the dark until use.

### recN Monoplex Immunoassay

All *rec*N conjugated bead sets, 229E *rec*N #78), NL63 *rec*N (#20), OC43 *rec*N3(#65), HKU1 *rec*N(#44), MERS-CoV *rec*N (#72,) SARS-CoV *rec*N (#26), pET *E. coli* Neg ctrl (#12 and His-BoNT Neg ctrl (#12) were brought to room temperature prior to performing the monoplex immunoassay. Beads were vortexed for 30 sec and then sonicated in a water bath sonicator (Branson ultrasonic cleaner, VWR International, West Chester, PA) for 20 sec. Each bead set was diluted in PBS (pH 7.4) to a final concentration of 100 beads/region/µl of PBS (pH7.4). A series of 2-fold dilutions (1:100–1:800) of positive and negative control sera were prepared in SurModics® Assay Diluent (cat#SM01–1000; SurModics, MN). In a black, 96 well, non-binding microtiter plate (Greiner Bio-One, Germany), 50 µl of diluted beads (100 beads/region/µl) and 50 µl of diluted control serum, were mixed using microtiter pipette. An additional 50 µl of PBS (pH 7.4) was added to each of the well of the plate. The plate was covered with aluminum foil and incubated for 2 h at room temperature with continuous shaking at speed 7 on titer plate shaker (Lab-Line Instruments, USA). Following incubation, the beads were separated using magnetic plate separator (*Luminex*^®^) for 60 sec and washed twice with 150 µl PBS (pH 7.4) using multichannel pipette. The washed beads were resuspended in 50 µL of PBS (pH 7.4) plus 50 µL of R- Phycoerythrin (R-PE) conjugated goat anti-human IgG (H + L) F (ab′)_2_ (cat#109-116-088; JacksonImmuno, USA) antibody. The detection antibody was prepared by diluting R-PE to 1:120 (v/v) in PBS. The plate was covered with aluminum foil and incubated for 30 min at room temperature with continuous shaking at speed 7 on titer plate shaker (Lab-Line Instruments, USA). Beads were separated and washed twice with 100 µL of PBS (pH 7.4) and finally resuspended in 100 µL of PBS (pH 7.4). The plate was read on *Luminex*^®^ MAGPIX^®^ system. The Mean Fluorescence Intensity (MFI) values of negative control antigen conjugated beads were subtracted from MFI values of their corresponding hCoV *rec*N antigens. The results were expressed as MFI of 200 beads/region/well.

### recN Multiplex Immunoassay

All *rec*N conjugated beads were brought to room temperature prior to performing the multiplex immunoassay. Beads were vortexed for 30 sec and then sonicated in a water bath sonicator (Branson ultrasonic cleaner, VWR International, West Chester, PA) for 20 sec. To prepare a working dilution, each bead set was diluted in PBS (pH 7.4) to a final concentration of 50 beads/region/µl of PBS (pH7.4). All diluted bead sets were then mixed into a single 15 ml polypropylene centrifuge tube in equal quantities. The 15 ml centrifuge tube was vortexed and sonicated as described above to create homogeneous mixture of all conjugated bead sets. All the test sera were diluted to an optimal dilution and tested in duplicate. To perform multiplexed bead immunoassay, 50 µl of homogeneous mixture of conjugated beads, 50 µl of diluted test serum sample and 50 µl of PBS (pH 7.4), were added to each of the well of the plate. The rest of the assay was carried out using the same steps as described above in the monoplex assay, except the results were expressed as MFI of 100 beads/region/well.

### Assay Sensitivity and Specificity

A panel of positive paired sera and negative paired sera from individuals with confirmed hCoV infection, was tested using multiplexed microsphere immunoassay (MMIA). Using rRT-PCR as the reference test, %sensitivity (%Se) and %specificity (%Sp) of the MMIA were calculated and plotted as Two Graph Receiver Operating Characteristic (TG-ROC) analysis plots. The intersections, of %Se and %Sp plots, were selected as the cut-off MFI for each *rec*N. The results were also plotted as ROC curve and the Area under the Curve (AUC) was calculated for each *rec*N to determine overall assay performance.

### Assay Reproducibility and correlation coefficient

A series of 2-fold dilutions (1:100–1:12800), of positive and negative controls of all six hCoVs, was prepared in SurModics® Assay Diluent (cat#SM01-1000; SurModics, MN) and tested using monoplex and multiplex immunoassay formats to determine correlation between two formats. The same panel was used in multiplex format for determining the cross-reactivity between hCoV *rec*N beads as well as within and between assay variations. Each sample of the panel was run in triplicate on two different plates. The trial was repeated after seven days using another set of conjugated beads.

### Statistical Analysis

IBM SPSS Statistics 21 software, was used to perform receiver-operating characteristic (ROC), area under the curve (AUC), and assay cut-off analysis. Linear regression analysis was performed using EXCEL (*Microsoft*^*®*^ Office 2010) to determine correlation coefficients and coefficient of variance for each antigen.

## Data Availability

Raw data will be made available upon request. Authors also agree to share reagents used in this manuscript.

## References

[1] Cabeca TK, Granato C, Bellei N (2013). Epidemiological and clinical features of human coronavirus infections among different subsets of patients. Influenza Other Respir. Viruses.

[2] Su S (2016). Epidemiology, Genetic Recombination, and Pathogenesis of Coronaviruses. Trends Microbiol..

[3] Zumla A, Chan JF, Azhar EI, Hui DS, Yuen KY (2016). Coronaviruses - drug discovery and therapeutic options. Nat. Rev. Drug Discov..

[4] Wevers BA, van der Hoek L (2009). Recently discovered human coronaviruses. Clin. Lab. Med..

[5] de Wit E, van Doremalen N, Falzarano D, Munster VJ (2016). SARS and MERS: recent insights into emerging coronaviruses. Nat. Rev. Microbiol..

[6] Dijkman R, van der Hoek L (2009). Human Coronaviruses 229E and NL63: Close Yet Still So Far. J. Formos. Med. Assoc..

[7] Zuwala K (2015). The nucleocapsid protein of human coronavirus NL63. PLoS One.

[8] Coleman CM, Frieman MB (2014). Coronaviruses: important emerging human pathogens. J. Virol..

[9] Morris-Cunnington MC (2004). A Population-based Seroprevalence Study of Hepatitis A Virus Using Oral Fluid in England and Wales. American J. of Epidemiol..

[10] Zhang Y (2014). A duplex recombinant viral nucleoprotein microbead immunoassay for simultaneous detection of seroresponses to human respiratory syncytial virus and metapneumovirus infections. J. Virol. Methods.

[11] Gao X (2015). Antibody against nucleocapsid protein predicts susceptibility to human coronavirus infection. J. Infect..

[12] Smits GP, van Gageldonk PG, Schouls LM, van der Klis FR, Berbers GA (2012). Development of a bead-based multiplex immunoassay for simultaneous quantitative detection of IgG serum antibodies against measles, mumps, rubella, and varicella-zoster virus. Clin. Vaccine Immunol..

[13] Basile AJ (2013). Multiplex microsphere immunoassays for the detection of IgM and IgG to arboviral diseases. PLoS One.

[14] Greiner M, Sohr D, Gobel P (1995). A modified ROC analysis for the selection of cut-off values and the definition of intermediate results of serodiagnostic tests. J. Immunol. Methods.

[15] Gardner IA, Greiner M (2006). Receiver-operating characteristic curves and likelihood ratios: improvements over traditional methods for the evaluation and application of veterinary clinical pathology tests. Vet. Clin. Pathol..

[16] Sun ZF, Meng XJ (2004). Antigenic cross-reactivity between nucleocapsid protein of severe acute respiratory syndrome (SARS) coronavirus and polyclonal antisera of antigenic group I animal coronaviruses: Implication for SARS diagnosis. J. Clin. Microbiol..

[17] Che X (2005). Antigenic cross-reactivity between Severe Acute Respiratory Syndrome-Associated coronavirus and human coronaviruses 229E and OC43. J. Infect. Dis..

[18] Haynes LM (2007). Recombinant protein-based assays for detection of antibodies to severe acute respiratory syndrome coronavirus spike and nucleocapsid proteins. Clin. Vaccine Immunol..

[19] Blanchard EG, Miao C, Haupt TE, Anderson LJ, Haynes LM (2011). Development of a recombinant truncated nucleocapsid protein based immunoassay for detection of antibodies against human coronavirus OC43. J. Virol. Methods.

